# Pharmacogenomic-based GWAS Meta-Analyses Coupled with Genetic and Epigenetic Liability Testing Connects Facial and Emotional Recognition Systems to Spectrum Disorders, Schizophrenia, Depression, and Anxiety Disorders

**DOI:** 10.2174/0113892010431102260107110422

**Published:** 2026-03-09

**Authors:** Kenneth Blum, Alexander P.L. Lewandrowski, Alireza Sharafshah, Albert Pinhasov, Kavya Mohankumar, Mark S. Gold, Brian Fuehrlein, Igor Elman, Catherine Dennen, Panayotis K. Thanos, Abdalla Bowirrat, David Baron, Edward J. Modestino, Nicole Jafari, Foojan Zeine, Keerthy Sunder, Milan T. Makale, Morgan P. Lorio, Jag Khalsa, Sergio Luis Schmidt, Rossano Kepler Alvim Fiorelli, Aviv Weinstein, Marco Lindenau, Kai-Uwe Lewandrowski, Óscar L. Alves, Shaurya Mahajan, Yatharth Mahajan, Rajendra D. Badgaiyan

**Affiliations:** 1 Department of Molecular Biology, Adelson School of Medicine, Ariel University, Ariel, 40700, Israel;; 2 Division of Addiction Research & Education, Center for Sports, Exercise, Psychiatry, Western University Health Sciences, Pomona, CA 91766-1854, USA;; 3 The Kenneth Blum Behavioral & Neurogenetic Institute, Austin, TX 78701, USA;; 4 Institute of Psychology, ELTE Eötvös Loránd University, H-1117 Budapest, Hungary;; 5 Department of Biological Sciences, Dornsife College of Letters, Arts and Sciences, 3616 Trousdale Pkwy, Los Angeles, CA 90089, USA;; 6 Cellular and Molecular Research Center, School of Medicine, Guilan University of Medical Sciences, Rasht 4144666949, Iran;; 7 Department of Psychiatry, Washington University School of Medicine, St. Louis, MO 63110, USA;; 8 Department of Psychiatry, Yale University, New Haven, CT, 06520, USA;; 9 Cambridge Health Alliance, Harvard Medical School, Cambridge, MA, 02139, USA;; 10 Department of Family Medicine, Jefferson Health Northeast, Philadelphia, PA, 19145, USA;; 11 Department of Pharmacology and Toxicology, State University of New York, SUNY, Buffalo, NY 14215, USA;; 12 Department of Psychiatry, Stanford University School of Medicine, Palo Alto, CA 94305, USA;; 13 Brain & Behavior Laboratory, Department of Psychology, Curry College, Milton, MA 02186, USA;; 14 Department of Applied Clinical Psychology, The Chicago School of Professional Psychology, Los Angeles, CA 60601, USA;; 15 Department of Health Science, California State University at Long Beach, Long Beach, CA 90815, USA;; 16 Department of Psychiatry, University of California, UC Riverside School of Medicine, Riverside, CA 92521, USA;; 17 Department of Radiation Medicine and Applied Sciences, University of California San Diego, La Jolla, CA 92093, USA;; 18 Advanced Orthopedics, Altamonte Springs, FL 32701, USA;; 19 Medical Consequences of Drug Abuse and Infections Branch, National Institute on Drug Abuse, National Institutes of Health, Bethesda, MD 20852, USA;; 20 Programa de Pós-Graduação em Neurologia, Universidade Federal do Estado do Rio de Janeiro, Rio de Janeiro 20270-004, Brazil;; 21 Department of Behavioral Sciences, Ariel University, Ariel, 40700, Israel;; 22 Division of Personalized Medicine, Center for Advanced Spine Care of Southern Arizona, Tucson, AZ 85712, USA;; 23 Department of Orthopaedics, Fundación Universitaria Sanitas, Bogotá 110131, Colombia;; 24 Department of Orthopaedic Surgery, University of Arizona, School of Medicine, Tucson, AZ 85724, USA;; 25 Department of Neurosurgery, Hospital Lusíadas, Porto, Portugal;; 26 Department of Psychiatry, Texas Tech University Health Sciences, School of Medicine, Midland, TX 79430, USA

**Keywords:** Facial physics, emotional expression, GWAS, meta-meta analysis, pharmacogenomics, ADHD, autism, schizophrenia, depression and anxiety

## Abstract

**Introduction:**

Facial and Emotional Recognition Systems are technologies that primarily use AI and machine learning to analyze various inputs like facial expression, speech, and physiological signals, to identify and classify human emotions and link them to a variety of epigenomic traits and states.

**Methods:**

We conducted a Meta-Meta Analysis via Pharmacogenomics (PGx) and Genome-Wide Association Studies (GWAS) across two separate manifestations, including facial physics and emotional expressions.

**Results:**

Applying GWAS datasets, 10 GWAS datasets were included, and following multiple filtrations, a GWAS Meta-Meta analysis led to a Secondary Gene List (SGL) of 586 members. Additionally, various in-depth silico analyses, such as Protein-Protein Interactions (PPIs), refined 300 genes into a unified network, then, by adding 10 GARS genes, 309 genes remained. A different analysis of PPIs uncovered 141 connected genes (Final Gene List: FGL); more precisely, we conducted a PGx-based approach on this FGL. Finally, 1,480 annotations were found, among them, 682 annotations were significant; thus, we considered the genes with at least one significant annotation and found 54 Pharmacogenes in FGL (PGx-FGL).

**Discussion:**

Through this in-depth analysis, we identified strong, significant top phenotypic roles for both DRD2 and BDNF linking genes in 48,780,906 subjects.

**Conclusion:**

Our PGx-based GWAS meta-meta-analyses, coupled with genetic and epigenetic liability testing, connected Facial and Emotional Recognition Systems to Spectrum Disorders (Attention-Deficit Hyperactivity Disorder: ADHD and Autism), Schizophrenia, Depression, and Anxiety. We propose that these findings could have heuristic therapeutic targeting potential and, as such, require intensive further clinical support.

## 1 INTRODUCTION

A facial recognition system [1] is a technology potentially capable of matching a human face from a digital image or a video frame against a database of faces. Such a system is typically employed to authenticate users via ID verification services and works by pinpointing and measuring facial features from a given image [2].

Development began with similar systems in the 1960s, beginning as a form of computer application. Since their inception, facial recognition systems have seen wider use in recent times on smartphones and in other forms of technology, such as robotics. Because computerized facial recognition involves measuring a human's physiological characteristics, facial recognition systems are categorized as biometrics. Although the accuracy of facial recognition systems as a biometric technology is lower than that of iris recognition, fingerprint image acquisition, palm recognition, or voice recognition, it is widely adopted due to its contactless process [3]. Facial recognition systems have been deployed in advanced human–computer interaction, video surveillance, law enforcement, passenger screening, decisions on employment and housing, and automatic image indexing [4, 5].

Facial recognition systems are employed throughout the world today by governments and private companies [6]. Their effectiveness varies, and some systems have previously been scrapped because of their ineffectiveness. The use of facial recognition systems has also raised controversy, with claims that they violate citizens' privacy, commonly make incorrect identifications, encourage gender norms [7, 8] and racial profiling [9], and do not protect important biometric data. The appearance of synthetic media, such as deepfakes, has also raised concerns about its security [10]. These claims have led to the ban of facial recognition systems in several cities in the United States [11]. Growing societal concerns led the social networking company Meta Platforms to shut down its Facebook facial recognition system in 2021, deleting the face-scanning data of more than one billion users [12, 13]. The change represented one of the largest shifts in facial recognition usage in the technology's history. IBM also stopped offering facial recognition technology due to similar concerns [14].

### 1.1 Practical Generalizations

Facial recognition is a technology used for the personal identification or grouping of individuals in images, both still and video. Face recognition is a type of computer vision that uses optical input to analyze an image—in this case, it looks particularly at faces that appear in the image. Facial recognition technology can be used as a building block to support other capabilities, such as face identification, grouping, and verification. Face recognition software can support many practical uses, both for businesses and for users at home. AI image recognition software analyzes the geometry of the face in an image by measuring the distances between key facial features, creating a unique facial template. The facial template is compared to a database of known, stored, or available faces. A determination is made if a match is found in the existing database. Face recognition technology is making the world safer, smarter, and more convenient—and as the need for advanced security grows, the potential uses for facial recognition continue to expand. Here are two basic ways this technology is frequently used: with face verification, two face templates are compared to determine whether they are a match. On a practical level, the purpose of a facial recognition algorithm is to evaluate whether two faces belong to the same person. Person identification uses photo-based face recognition to compare a detected face against a database of people in order to determine identity. In this expert opinion, we aim to explore the potential of coupling Facial Emotion Recognition (FER) with an accurate genetic test to predict not only psychopathology but also preaddiction.

### 1.2 Facial Identification in Psychopathology

FER is the ability to recognize and discriminate basic emotional states from facial expressions [15]. Facial emotion recognition deficits have been consistently demonstrated in patients with severe mental disorders [16]. Moreover, FER is the ability to recognize and discriminate basic emotional states from facial expressions. In one study, Nejati *et al*. found that relevant cortical areas for improvement of FER with Non-Invasive Brain Stimulation (NIBS) were the dorsolateral Prefrontal Cortex (dlPFC), the ventromedial Prefrontal Cortex (vmPFC), the right Temporoparietal Junction (rTPJ), the posterior Superior Temporal Sulcus (pSTS), the left pre-Supplementary Motor Area (pre-SMA), and the somatosensory area [15].

Importantly, the vmPFC has been implicated in a variety of social, cognitive, and affective functions that are commonly disrupted in mental illness. One line of research indicates that the vmPFC is critical for the representation of reward- and value-based decision making, through interactions with the ventral striatum and amygdala [17]***.*** A second line of research demonstrates that the vmPFC is critical for the generation and regulation of negative emotion, through its interactions with the amygdala, bed nucleus of the stria terminalis, periaqueductal gray, hippocampus, and dorsal anterior cingulate cortex [17]**.** A third line of research highlights the importance of the vmPFC across multiple aspects of social cognition, such as facial emotion recognition, theory-of-mind ability, and processing self-relevant information, through its interactions with the posterior cingulate cortex, precuneus, dorsomedial PFC, and amygdala [17]**.** Along these lines, a meta-analytic data revealed distinct subregions within the vmPFC that correspond to each of these three functions, as well as the associations between these subregions and specific psychiatric disorders (depression, posttraumatic stress disorder, addiction, social anxiety disorder, bipolar disorder, schizophrenia, and attention-deficit/hyperactivity disorder).

### 1.3 ADHD

 Autism Spectrum Disorder (ASD) and Attention-Deficit Hyperactivity Disorder (ADHD) have been associated with alterations in Facial Affect Recognition (FAR). In terms of psychopathology and the importance of FER has been the focus of some researchers, and it is indeed noteworthy that Staff *et al*. reported that by using Morphed Facial Emotion Recognition Task (MFERT), whereby teachers and parents rated social and emotional problems on the Strengths and Difficulties Questionnaire, repeated measures analysis of variance revealed that the ADHD group showed poorer emotion recognition accuracy compared to controls across emotions. Interestingly, multiple regression analyses within the ADHD group showed that emotion recognition accuracy was inversely related to social and emotional problems, but not to prosocial behavior. Not only children with an ADHD diagnosis, but also children with subthreshold ADHD experience impairments in facial emotion recognition. This impairment is predictive of social and emotional problems, which may suggest that emotion recognition may contribute to the development of social and emotional problems in these children [18].

In another study, patients with ADHD had a significantly lower number of correct answers in the child and adolescent Reading the Mind in the Eyes Task (RMET) and in the Benton Face Recognition Test (BFRT) than the healthy controls. Among the ADHD subtypes, the hyperactive/ impulsive subtype had a lower number of correct answers in the RMET than the inattentive subtypes, and the hyperactive/ impulsive subtype had a lower number of correct answers in the short and long form of BFRT than the combined and inattentive subtypes. Male and female patients with ADHD did not differ significantly with respect to the number of correct answers on the RMET and BFRT [19]**.** In contrast, others reported that children with ADHD have similar levels of trait and state empathy skills and facial expressions as healthy controls, and CPs negatively affect their empathy skills [20]. In addition, it is noteworthy that Schizophrenia and ADHD both affect facial affect recognition, and scanpaths to facial expressions are impaired in schizophrenia, with emerging empirical evidence showing that facial affect recognition is also impaired in ADHD. Brain imaging studies show reduced activity in the medial prefrontal and limbic (amygdala) brain regions required to process emotional faces in schizophrenia, but suggest more localized loss of activity in these regions in ADHD. As amygdala activity in particular has been linked to effective visual scanning of face stimuli, it is postulated that condition-specific breakdowns in these brain regions that subserve emotional behavior might manifest as distinct scanpath aberrations in facial expressions of emotion in schizophrenia and ADHD [21]. It is noteworthy that an ADHD Diagnostic System (ADS) and a computerized Continuous Performance Test (CPT) were used to evaluate attention performance. Subjects with ADHD showed significantly lower scores of contextual understanding than the controls (t = -2.569, df = 67, P = 0.012). Attention deficits in boys with ADHD seemed to account for their difficulty in recognizing facial expressions of emotion [22]**.** Interestingly, using facial recognition and Saccadic movement deficiencies reveals that ADHD individuals are more easily distracted by a task-irrelevant gaze direction. Taken together, these results suggest that tasks requiring both response inhibition (antisaccade) and gaze-emotion recognition might be useful for developing a diagnostic test for discriminating between adults with ADHD and healthy adults [23]. Moreover, it was reported by Williams *et al*. ADHD subjects showed a profile of emotion-related impairment: higher depression and anxiety, deficits in identifying threat-related emotional expressions in particular, and alterations in Event-Related Potentials (ERPs). There was a pronounced reduction in occipital activity during the early perceptual analysis of emotional expression (within 120 msec), followed by an exaggeration of activity associated with structural encoding (120-220 msec) and subsequent reduction and slowing of temporal brain activity subserving context processing (300-400 msec) [24]. Emotional facial stimuli are important social signals that must be perceived and recognized in order to make appropriate decisions and responses in everyday communication. The ability to voluntarily guide attention to perceive and recognize emotions, and to react to them, varies largely across individuals, and has a strong genetic component. Two key genetic variants of the catecholamine system that have been related to emotion perception and attention are the catechol-O-methyl transferase genetic variant (COMT Val158Met) and the α2A-receptor gene promoter polymorphism (ADRA2A C-1291G) accordingly. In simple visual detection, the ADRA2A C-1291G G-allele leads to slower detection of a highly arousing face (scheming), which is modulated by each additional COMT Val158Met Met-allele and male sex predicting faster responses. The combination of G-allele, Met-allele and male sex also predicts higher perceived negativity in sad faces. No effects of C-1291G, Val158Met, and sex were found on verbal emotion recognition [25]. In fact, one review pointed out that 58% of retrieved articles reported significantly decreased emotion recognition accuracy among individuals with ADHD relative to neurotypical peers, with either global or emotion-specific differences identified [26].

### 1.4 AUTISM

 Emotion Recognition (ER) deficit in ASD is widely accepted, and a variety of research has been done to assess the deficit in ER under various conditions and intervention research to augment emotion recognition, mostly in children with ASD. The results show mixed findings: the majority of work indicates a deficit in facial emotion recognition in ASD, but under certain stimuli and conditions, ASD performs well in facial emotion recognition. Moderating factors identified in one review of ER included age, IQ, comorbidity, task paradigm, and cultural factors [27]. Furthermore, it has been found that reduced activation in the Fusiform Gyrus (FG), amygdala, Superior Temporal Sulcus (STS), and Prefrontal Cortex (PFC) contributes to difficulties in processing facial cues, while increased reliance on the PFC for facial recognition tasks imposes a cognitive load. Additionally, disrupted functional and structural connectivity between these regions further exacerbates facial recognition challenges [28]. Recently, another study showed that Autistic individuals performed as well as non-autistic ones in terms of recognition accuracy/sensitivity across measures and emotional categories, but took longer to do so [29]**. **In fact, Kirsch *et al*. showed an unexpected dissociation between behavior and brain response: While ASD affects facial recognition performance, only co-occurring alexithymia modulates corresponding social brain activations. Although the study does not support the alexithymia hypothesis at the behavioral level, it highlights the complex relationship between ASD and co-occurring alexithymia, emphasizing the significance of co-occurring conditions in understanding emotion processing in ASD [30]. Importantly, other results suggest that ASD engages core face-processing areas and underscores the role of the fusiform face area in facial emotion processing, along with more insights into the neural processing of facial emotions [31]. In addition, others found better FER consistent with higher social anxiety and better updating skills in ASD, while with lower social anxiety in Specific Learning Disorders (SLD). The authors suggested that clinical practice should focus on coping strategies in autistic youth who could feel anxiety when facing social cues, and on self-efficacy and social worries in SLD [32]. Executive functioning should also be addressed to support social learning in autism. In fact, linear regression models revealed that (1) handedness predicted FEP hemispheric choice bias, (2) the attention switching Autism-spectrum Quotient subscale predicted FEP hemispheric reaction time bias, and (3) the imagination Autism-spectrum Quotient subscale predicted FEP hemispheric reaction time bias for males, but not females [33]. It is noteworthy that Autism Spectrum Condition (ASC) is characterized by atypical attention to eyes and faces, but the onset and impact of these atypicalities remain unclear. One prospective longitudinal study examined face perception in infants who develop ASC (N = 22, females = 5, 100% White) compared with typically developing infants (N = 131, females = 65, 55.6% White), tracking social-cognitive and ASC development through age seven. Reduced interest in direct gaze and eyes during infancy correlated with atypical development of adaptive behavior at age four and theory of mind at age seven. Principal component analyses revealed less integrated processing of facial features and eye-gaze information in ASC infants, potentially impacting their childhood social functioning. These findings highlight the intertwined nature of social-cognitive development and ASC [34]. Indeed, another study found that infants at 24 months with a High Likelihood (HL) for autism, oriented towards eyes more slowly after being guided to the mouth. After being guided to the eyes, HL infants' secondary fixation fell less in the eye region, and their latency to orient away from the eyes also tended to be shorter. This investigation contributes to the theoretical understanding of the atypical face-scanning pattern in autistic people and its related underlying mechanisms. Furthermore, this study provides important insights into the development of early screening tools and intervention protocols for autistic people [35]. Moreover, Hill *et al*. revealed that increased facial emotion processing-related connectivity across theta (4 to 7 Hz) and beta (13 to 30 Hz) frequencies correlated positively with higher Social Responsiveness Scale (SRS-2) scores, predominantly in right-lateralized (theta) and bilateral (beta) cortical networks. Additionally, a steeper 1/f-like aperiodic slope (spectral exponent) across fronto-central electrodes was associated with higher Social Responsiveness Scale (SRS)-2 scores. Greater aperiodic-adjusted theta and alpha oscillatory power further correlated with both higher SRS-2 scores and steeper aperiodic slopes. These findings underscore important links between facial emotion processing-related brain dynamics and autistic traits in typically developing children [13].

Our strategy involved exploring the GWAS Meta-Meta analysis of FER, a genetic network between facial physics and emotional phenotypes. The aim of this study was to investigate the plausible genetic roles as a molecular bridge between facial physics and emotional phenotypes. To accomplish this aim, we designed a serial analytical investigation based on our newly published strategy [36-38]. Briefly, the main concept of this strategy establishes reliable logic by searching the raw data in the GWAS database, refining the datasets, merging them to build metadata, and then generating an innovative gene list that will predict new connections, genetic markers, and potential signaling pathways.

## 2 MATERIALS AND METHODS

### 2.1 Databases, Datasets, and Metadata

A comprehensive search was conducted on GWAS data to find updated genomic information concerning facial emotion and morphology with psychological emotions, including ADHD, Autism Spectrum Disorder, Schizophrenia, Depression, and Anxiety, via a SNP-trait association filtering strategy. According to GWAS catalog (https://www.ebi.ac.uk/gwas/home), 12 GWAS IDs found were based on the aforementioned terms including “facial emotion recognition measurement” [catalog ID: EFO_0008329]; “emotional symptom measurement” [catalog ID: EFO_000780^3^]; “facial attractiveness measurement” [catalog ID: EFO_0009892]; “facial morphology trait” [catalog ID: OBA_VT00037^43^]; “facial morphology” [catalog ID: EFO_00047^43^]; “amygdala volume” [catalog ID: OBA_20500^34^]; “attention deficit hyperactivity disorder” [catalog ID: EFO_0003888]; “schizophrenia” [catalog ID: MONDO_0005090]; “depressive disorder” [catalog ID: MONDO_0002050]; “anxiety” [catalog ID: EFO_0005230]; “anxiety disorder” [catalog ID: EFO_0006788]; and “autism spectrum disorder” [catalog ID: EFO_00037^56^]. Thus, raw datasets included 12 traits. In summary, by integrating all inputs, multiple cutoffs were considered by ordering the unique annotations from the most significant p-values (all annotations with p-value >5E-08 were removed), discarding pseudogenes and RNA genes, and then deleting all duplicated genes.

Notably, computational analyses linking the mapped genes identified by various GWAS in each dataset (Primary Gene List: PGL) were predominantly based on a separate GWAS Meta-Analysis. All the remaining mapped genes generated cumulative mapped genes from all datasets (Secondary Gene List: SGL), which were then validated by a GWAS Meta-Meta Analysis. Following the initial check, two GWAS datasets were removed due to a lack of significant annotations with a p-value lower than 5E-08 (EFO_0008329 and EFO_0009892). Thus, we selected 10 datasets through the abovementioned Catalog IDs (CIDs), including “emotional symptom measurement” [catalog ID: EFO_0007803 as Meta^1^]; “facial morphology trait” [catalog ID: OBA_VT0003743 as Meta^2^]; “facial morphology” [catalog ID: EFO_0004743 as Meta^3^]; “amygdala volume” [catalog ID: OBA_2050034 as Meta^4^]; “attention deficit hyperactivity disorder” [catalog ID: EFO_0003888 as Meta^5^]; “schizophrenia” [catalog ID: MONDO_0005090 as Meta^6^]; “depressive disorder” [catalog ID: MONDO_0002050 as Meta^7^]; “anxiety” [catalog ID: EFO_0005230 as Meta^8^]; “anxiety disorder” [catalog ID: EFO_0006788 as Meta^9^]; and “autism spectrum disorder” [catalog ID: EFO_0003756 as Meta^10^]. Meta-Meta analysis of Meta1 to Meta10 was also conducted to reveal the significance and cumulative Effect Size (ES) according to p-value, z-score, and OR.

Using inclusion/exclusion criteria after launching separate GWAS meta-analyses for each GWAS dataset via the CMA3 tool, we successfully obtained 10 separate metadata (Meta1-Meta10). Evidently, the different types of inclusion criteria were as follows: unique GWAS dataset, published papers, GWA studies with a clear number of subjects, p-value < 5E-08 (as GWAS consensus threshold of significance), having calculated OR/Beta (CI95%) numbers, papers reported mapped genes and their related Single Nucleotide Polymorphisms (SNPs). It should be noted and clarified herein that the selection of p-values in our analysis passed a two-step process. First, all of the data in the GWAS datasets with p-values lower than 5E-08 were included, and the rest of the data with p-values > 5E-08 were excluded; in the second step, among the SNPs of the remained studies, the weakest p-values (which are less than 5E-08) were designated to be included as the p-value entry for the meta- and meta-meta-analyses. For clarity, it should be noted that in studies having several SNPs in the dataset, the included OR value in CMA3 from a particular study was considered based on the weakest (the most cautious) p-value related to that SNP. Inversely, the exclusion criteria were as follows: duplicated reports, unpublished papers (Preprint or under review versions), p-value > 5E-08, lost OR/Beta and CI95%, articles without specific mapped genes, and unspecified SNPs. Moreover, regarding both our Meta-analysis and Meta-Meta Analysis of relevant GWAS data using the CMA3 tool, which analyzed both random and fixed effects sizes, we followed this directive path: correlation > computed effect size > p-value and sample size for correlation. Finally, the entry data were as follows: total sample size, best-reported p-values (2-tailed), and positive effect direction (due to the nature of GWAS associations).

Following performing the GWAS Meta and GWAS Meta-Meta Analyses, all significant mapped genes were extracted from all datasets and combined into a single list (SGL). This Pre-Secondary Gene List (PreSGL) consisted of a large file (raw data) including duplications, RNA genes, pseudogenes, and protein-coding genes. As such, in a final attempt to filter the resultant significant gene list, we deleted duplications, RNA genes, and pseudogenes. Following this critical step, we reached a polished list of protein-coding genes. Other analyses were then performed on this SGL. All details and related references are listed in Table **1**.

## 3 GWAS META, GWAS META-META, IN-DEPTH SILICO, AND SYSTEMS BIOLOGY ANALYSES

Successfully, our innovative investigative strategy has recently been published and replicated in a number of prestigious journals based on our previously published papers [44-47]. In summary, by initially (first step) utilizing GWAS Meta and GWAS Meta-Meta Analyses, verifying the necessary statistical support, we proceeded to the next steps. Notably, to our best knowledge, this work represents the first GWAS Meta-Meta analysis method reported in the literature (PubMed + Google Scholar), suggesting a new strategy for computational AI neuroscience and genomic aspects.

To reiterate for accuracy, our strategy encompassed additional in-depth silico investigations, systems biology analyses, and Pharmacogenomics (PGx) approaches. We performed Protein-Protein Interactions (PPIs) using a two-step STRING-MODEL to help identify the interconnected members (Proteogenomics). This strategy helped us identify the most interconnected genes as potential candidates linked to facial physics and emotional phenotypes. Afterward, following this line of examination, we established what we term a new *“Secondary Gene List “*(SGL), which played a linking role between 10 PGLs and the “*Final Gene List*” (FGL).

Consequently, FGL further defined a more in-depth silico analysis by performing another PPIs in a STRING-MODEL. Additonally, we further analyzed both GMIs and TF-Gene interactions (TFGIs) via miRTargetLink2.0 and NetworkAnalyst [using R package], respectively. This was further followed by an Enrichment Analysis via Enrichr by subsequent pathway (s), ontology, and Disease-Drug Analysis. All pertinent details and the strategy of analysis are described in Table **1**. To enhance comprehension of this multipart methodology, an illustrated flowchart for this strategy is provided in Fig. (**1**).

### 3.1 Statistical Evaluations

 The expected number of edges in the STRING-MODEL is the number of edges that would be expected if the nodes were chosen at random. A small PPI enrichment p-value indicates that the nodes are not random and that the observed number of edges is significant. Two popular node centrality measures provided by NetworkAnalyst are degree and betweenness. High-degree nodes are regarded as the hubs of the network. High-degree nodes act as network hubs because, even with a low degree, they have high betweenness if they are situated between two dense clusters. The number of routes that go through a node is called its betweenness when considering the pairwise shortest pathways between each node in the network. If a node is situated between two dense clusters, it will have a high betweenness even if its degree is low. Furthermore, NetworkAnalyst uses gene expression values as edge weights when searching for modules. In addition, the weights were evaluated by the square of the mean absolute log fold changes of the two adjacent nodes. Non-seed proteins are assigned pseudo-expression values equal to 1/10 of the minimal absolute log fold changes of the seed proteins to prevent zero-weight mistakes for non-seed proteins during program functioning. The software prioritizes finding modules with additional seed proteins (shorter distances) by assigning bigger weights to seed proteins. The Wilcoxon rank-sum test of the "internal" and "external" degrees was performed to compute the p-value of a specific module. The p-value, q-value, rank (Z-score), and combined score are the four metrics that Enrichr uses to report enrichment results. It should be noted that we did not include the combined score for p-value, q-value, and OR (common scores in the biological reports). The hypergeometric test or Fisher's exact test, two common statistical techniques that employ the majority of enrichment analysis tools, were used to assess the p-value. A binomial distribution and independence for the likelihood of any gene belonging to any set were considered by this binomial percentage test. The q-value was an altered p-value that has been corrected for multiple hypothesis testing based on the Benjamini-Hochberg procedure [48]. Meta-Meta analysis was performed using Comprehensive Meta-Analysis (CMA3) based on Fisher’s z-transformed correlation coefficients from the 10 independent meta-groups. For each study, the summary correlation (r), 95% confidence interval, z-value, and p-value were extracted. Both fixed-effects and random-effects models were fitted. Heterogeneity was assessed using Cochran’s Q, degrees of freedom (df), I², τ, and τ².

## 4 RESULTS

### 4.1 GWAS Meta-Analysis and Meta-Meta Analysis Results

A total of 19,792 associations and 3,704 repeated studies across various ethnicities were extracted from the 12 GWAS traits as raw data. Following preparation of data for GWAS Meta analyses, the results indicated that two datasets, including Facial Emotion Recognition measurement (EFO_0008329) and Facial Attractiveness Measurement (EFO_0009892), could not be included in the next step due to a lack of annotations with p-value lower than 5E-08, protein-coding genes, or having intergenic or synonymous SNPs. Thus, after primary filtration, 10 datasets (Meta1-Meta10) were included, and the final outcomes were derived from these datasets. There were 220 unique studies, 2,977 mapped genes, and 48,780,906 multi-ethnic individuals calculated by adding all the refined data from every 10 Metadata (Table **2**). The details of screening, filtering, and the number of studies are presented in the PRISMA diagram (Fig. **2**).

Our Meta-analysis results for every Meta group were as follows: p-value and Correlation(r) scores for Meta1-Meta10 were 1E-10 and 0.0044, 1E-10 and 0.0093, 2E-04 and 0.031, 1E-10 and 0.19, 1E-10 and 0.0023, 1E-10 and 0.0021, 1E-10 and 0.0014, 1E-10 and 0.0073, 1E-10 and 0.0028, and finally, 1E-10 and 0.0039, respectively. Additionally, both random and fixed-effect models indicated p-values lower than 1E-10. In addition, the fixed model showed a Z-value of 16.40 and an r of 0.0023, and the random model indicated a Z-value of 6.94 and an r of 0.0045. Fig. (**3**) illustrates the Meta-Meta Analysis results in a Forest Plot with Favored regions (correlation with 95% CI) and relative weights (Fig. **3**).

The combined analysis showed: Q = 121.126, df = 9, p < 1×10⁻¹⁶; I² = 92.57%; τ ≈ 0.02, τ² ≈ 0.0004, which rounded to 0.00 in CMA tool. Importantly, although I² was high, τ² was essentially zero, indicating very small absolute heterogeneity despite large relative heterogeneity. This pattern is expected in meta-analyses with extremely precise estimates (large GWAS samples), where even tiny study-to-study differences inflate Q and I² while τ² remains negligible. Concerning τ², which was nearly 0, the random-effects model approaches the fixed-effects solution; therefore, both models are reported without favoring one over the other. Although these pooled correlations are statistically significant due to the very large sample sizes, their magnitude is extremely small (< 0.005). Heterogeneity statistics were: Q = 121.126 (df = 9, p < 10⁻¹⁶), I² = 92.57%, and τ² ≈ 0.00. This combination indicates high relative heterogeneity (inflated by very precise studies) but virtually zero absolute between-study variation.

### 4.2 Protein-Protein Interactions (PPIs)

As acknowledged in the method section, non-coding transcripts, intergenic, and synonymous variants were removed, along with RNA-coding genes and pseudogenes. According to an acceptable threshold in the GWAS consensus (p-value < 5E-08), 586 genes were included for further analysis. The STRING-MODEL of PPIs showed that 300 genes remained in the primary PPI step (SGL). STRING-MODEL scores were as follows: PPI enrichment p-value < 1E-16; average node degree = 1.46; average local clustering coefficient = 0.301.

## 5 SECONDARY PROTEIN-PROTEIN INTERACTIONS (PPIS)

The SGL contained 300 combined genes. Finally, after two-step PPIs via STRING-MODEL, we identified 141 fully connected genes (FGL) resulting from the PGL (586 genes). Additionally, the second STRING-MODEL scores were as follows: PPI enrichment p-value = 1.E-16; average node degree = 2.24; average local clustering coefficient = 0.617. Notably, the remaining analyses performed herein will be based on this FGL (141 genes) (Fig. **4**).

### 5.1 Gene-miRNA Interactions

Applying miRTargetLink2.0, a Concentric model predicted the potential GMIs among the FGL members (141 genes). GMIs indicated involvement of GARS genes (SLC6A4 and MAOA) in the network. Clearly, SLC6A4 and MAOA interacted with *BDNF* via hsa-miR16-5p and hsa-miR-22-3p (Fig. **5**).

### 5.2 TF-gene Interactions

According to a Fruchterman-Reingold model, results from the NetworkAnalyst tool exposed a connected network among the FGL members and Transcription Factors (TFs). *DRD2* and *BDNF* were involved in this network (Fig. **6**). Thus, this network validated our primary idea about the potential roles of *DRD2* and *BDNF* in the complex interactions of molecular pathways and networks between Facial Physics and emotional expressions.

### 5.3 Enrichment Analysis (EA)

We designed an enrichment analysis for systems biology investigation(s), which in turn, was separated into three main classifications, including Pathway Analysis (PA), Ontology Analysis (OA), and Disease-Drug Analysis (DDA) by the Enrichr tool. At this level, we ranked the FGL members along with their adjusted p-values (q-values) verified from different databases, such as Reactome, KEGG, GO, GeDiPNET, and DisGeNET.

### 5.4 Pathway Analysis (PA)

Table **3** indicates PA of FGL members as a subcategorized analysis of Enrichment Analysis. Essentially, after combining and prioritizing the candidate genes according to the most significant q-values, results from the Reactome database predicted that the Neural System pathway is the most significant pathway (q-value = 8.21E- 15; p-value = 1.15E-17). KEGG, on the other hand, predicted Dopaminergic synapse involvement as the bestscored pathway (q-value = 2.41E-13) (Table **4**).

### 5.5 Gene Ontology Analysis

Table **4** indicates our GO analysis, emphasizing the significance of Calcium Channel Complex (GO:0034704) as the most significant cellular component (q-value = 2.07E-07; p-value= 2.46E-09). Furthermore, Voltage-Gated Calcium Channel Activity (GO:0005245) (q-value= 2.06E-06) was the top-scoring in the Molecular Function. Interestingly, Dopamine Metabolic Process (GO:0042417) was the most significant Biological Process (q-value = 4.11E-06).

### 5.6 Disease-Drug Assessments (DDAs)

To identify the potential predictive manifestations of FGL members, we performed another Enrichment Analysis on the basis of disease/drug-based databases applying GeDiPNet and DisGeNET. Both analyses revealed that the most significant manifestation predicted to be at the highest risk of incidence is Schizophrenia (q-value= 8.76E-47 and q-value= 1.65E-32, respectively) (Table **5**). Importantly, *DRD2 and BDNF* were identified as the genes involved in these manifestations, as were *DRD1, DRD3, DRD4, MAOA, COMT, SLC6A3,* and *SLC6A4.* Amazingly, in all of the predicted manifestations (except Intelligence, Atrial Fibrillation, and Breast Carcinoma), *DRD2* and *APOE* genes or at least one of them were present (Table **5**).

### 5.7 PGx of FGL

The rationale behind this analysis stems from the idea that Pharmacogenomics conceptualizes that there are strong signaling pathways in which genes and drugs, as receptors and ligands, are involved. Accordingly, if a member in FGL has at least a significant pharmacogenomic association, then it would enable the precise selection of them for future investigations. Briefly, for comprehension purposes, Pharmacogenes display additional biomedical evidence and are more reliant on *in silico* predictions, possibly leading to a new concept in Personalized Medicine treatment (PGx-based Personalized Medicine).

Along these lines, we checked all 141 members of FGL and found a total of 1,480 annotations, of which 682 annotations were significant; thus, we considered the genes with at least one significant annotation and found 54 Pharmacogenes in FGL. The final PGx-based FGL members (PGx-FGL) included *DRD2, BDNF, MTHFR, COMT, SLC6A4, DRD3, ESR1, TCF7L2, CACNA1C, DRD1, GRM3, SLC6A3, NRXN1, CACNB2, KCNJ6, F2, ARID5B, BCL11A, CDH13, DRD4, SEPTIN3, ASTN2, CACNA2D3, ESR2, MAOA, CACNA1E, FOXO3, GABRB1, GRID2, NRG1, NTRK2, RPTOR, RYR2, ADCY2, ADGRV1, CACNA1D, CACNA1I, CTNNA2, CTNNA3, DLG2, DPP6, EHMT2, ERBB4, KLC1, KMT2E, MAML3, NCAM1, NOTCH4, PRKG1, RASSF1, RBFOX1, RBX1, RHOA, and VTI1A*.

### 5.8 Epigenomics of FGL

Methylation sites (CpGs) that are created or eliminated by Single-Nucleotide Polymorphisms (SNPs) are of particular importance. These CpG-SNPs can vary simultaneously in their DNA sequence and methylation state, providing information beyond what either data type provides on its own. However, methylation-dependent association signals at CpG-SNPs are not detected in GWAS and are only weakly identified in standard Methylome-Wide Association Studies (MWAS). Therefore, a dedicated CpG-SNP–focused analytical approach is required to reliably capture these signals [49]. Accordingly, we extracted all refined SNPs related to the PGx-FGL from all 10 metadata files and found 197 unique SNPs. In the next step, we searched all these SNPs in the dbSNP database and checked their CpG site status (Gain/Loss). We identified 37 genes related to 80 potential CpG-SNPs (42 Gain and 38 Loss). Surprisingly, DRD2 and BDNF had CpG-SNPs in this step as well (Table **6**).

## 6 DISCUSSION

Interestingly, we found that 9 of these denoted genes are found in the GARS test. Importantly, *DRD2* and *BDNF* are present in the PGx-FGL, which confirms our previous findings, and we hereby propose that future work should focus on these genes, their related genetic interactions, and also other epigenetic and expression impacts and biomarkers. Moreover, it is important to point out that both *DRD2 and BDNF* were among the most important linking genes. DRD2 was present in PGL2 [emotional symptom measurement_EFO_000780^3^], PGL9 [depressive disorder_MONDO_00020^50^], and PGL11 [anxiety disorder_EFO_0006788], and BDNF was present in PGL2 [emotional symptom measurement_EFO_000780^3^] and PGL7 [attention deficit hyperactivity disorder_FO_0003888].

Certainly, there is a plethora of literature evidence suggesting the important role of dopaminergic dysfunction in depressive disorders (11,806 PUBMED-listed articles as of 5-1-2025). Specifically, the dopamine hypothesis of schizophrenia and the emphasis on other neurotransmitters, most notably norepinephrine, serotonin, and acetylcholine, in the pathogenesis of depression, have focused attention away from substantial evidence implicating dopamine in affective disorders. The clinical evidence includes alterations in depressive symptoms with aging (concomitant with possible changes in dopamine metabolism), potential dopaminergic involvement in several subtypes of depression, similarities between some of the symptoms of Parkinson's disease and those of depression (including psychomotor retardation and diminished motivation), and potential dopaminergic abnormalities in seasonal mood disorder. The biochemical evidence in patients with depression derives from studies of Homovanillic acid, a dopamine metabolite, indicating diminished dopamine turnover. In addition, there is a considerable amount of pharmacological evidence regarding the efficacy of antidepressants with dopaminergic effects in the treatment of depression. It is well-established that dopamine contributes significantly to the pathophysiology of depression. However, the role of dopamine in this syndrome must be understood in the context of existing theories involving other neurotransmitters and genes like BDNF, which may act independently, and interact with dopamine and other neurochemicals, to contribute to depression [50].

In fact, most recently, the RDS Consortium published an article pointing out that in the United States, approximately 1 in 5 children experience comorbidities with mental illness, including depression and anxiety, which lead to poor general health outcomes. Adolescents with substance use disorders exhibit high rates of co-occurring mental illness, with over 60% meeting diagnostic criteria for another psychiatric condition in community-based treatment programs. Comorbidities are influenced by both genetic (DNA antecedents) and environmental (epigenetic) factors [36]. In this regard, Lewandrowski *et al*. [36] developed and performed a study evaluating the significant impact of psychiatric comorbidities on individuals' lives, to uncover common mechanisms through a Genome-Wide Association Study (GWAS) meta-meta-analysis. GWAS datasets were obtained for each comorbid phenotype, followed by a GWAS meta-meta-analysis using a significance threshold of *p* < 5E-8 to validate the rationale behind combining all GWAS phenotypes. The combined and refined dataset was subjected to bioinformatic analyses, including Protein-Protein Interactions and Systems Biology. PGx annotations for all potential genes with at least one PGx were tested, and the genes identified were combined with the Genetic Addiction Risk Severity (GARS) test, which included 10 genes and 11 Single Nucleotide Polymorphisms (SNPs). The STRING-MODEL was employed to discover novel networks and Protein-Drug interactions. ASD was identified as the top manifestation derived from the known comorbid interaction of anxiety, depression, and ADHD. The STRING-MODEL and Protein-Drug interaction analysis revealed a novel network associated with these psychiatric comorbidities. The findings suggest that these interactions are linked to the need to induce "dopamine homeostasis" as a therapeutic outcome. This study provides a reliable genetic and epigenetic map that could assist healthcare professionals in the therapeutic care of patients presenting with multiple psychiatric manifestations, including anxiety, depression, and ADHD. The results highlight the importance of targeting dopamine homeostasis in managing ASD linked to these comorbidities. These insights may guide future pharmaco-genomic interventions to improve clinical outcomes in affected individuals and tend to support the current investigation.

It is to be further noted that the pathogenesis of multiple depression is not fully clear, but its occurrence can be related to biological or environmental pathways, among which Brain-Derived Neurotrophic Factor (BDNF) can unequivocally act on two downstream receptors, Tyrosine Kinase Receptor (TrkB) and the p75 neurotrophin receptor (p75NTR), then affect the related signal pathways, affecting the occurrence and development of depression. Accumulating studies have revealed that *BDNF*-related pathways are critical in the pathophysiology of depression, and their interaction can further influence the efficacy of depression treatment [51]. In addition, Brain-derived neurotrophic factor is a key factor in the adaptation to stress and in the avoidance of a social stress behavioral response. Recent studies have shown that brain-derived neurotrophic factor expression in stressed mice is brain-region specific, particularly within the corticolimbic system, including the ventral tegmental area, nucleus accumbens, prefrontal cortex, amygdala, and hippocampus [52].

Han *et al*. (2025) found that stress-induced alterations in brain-derived neurotrophic factor levels are likely related to the nature, severity, and duration of stress, especially in the above-mentioned brain regions of the corticolimbic system. Therefore, *BDNF* might be a biological indicator regulating stress-related processes in various brain regions and affecting schizophrenia, ADHD, Post-Traumatic Stress Disorder (PTSD), Autism, depression, anxiety, and other RDS addictive behaviors, both drug and non-drug behavioral addictions [52-58].

Finally, it is noteworthy that PFOCRummage (https://pfocrummage.maayanlab.cloud/about) found a similar paper based on our 141 FGL. A study by Gupta *et al*. investigated 171 genes titled “Cerebrospinal Fluid Proteomics for Identification Of α2-Macroglobulin as a Potential Biomarker to Monitor Pharmacological Therapeutic Efficacy in Dopamine Dictated Disease States of Parkinson’s Disease and Schizophrenia,” which had 17 overlapped genes with our FGL (p-Value= 1.73E-12; adjusted p-Value= 8.48E-8). Specifically, they found that Protein profiles were delineated in Cerebrospinal Fluid (CSF) from Parkinson's disease patients, neurological controls, and schizophrenia patients, respectively. Ten proteins that were identified showed a linear relationship across the dopaminergic spectrum. α-2-Macroglobulin was shown to have high statistical significance in inter-group comparisons on validation studies using ELISA [59].

## CONCLUSION

Finally, via this in-depth analysis, we identified strongly significant top phenotypic roles for both *DRD2* and BDNF linking genes in 48,780,906 subjects. Moreover, our Pharmacogenomic-based GWAS Meta-Meta analyses, coupled with genetic and epigenetic liability testing, connected Facial and Emotional Recognition Systems to Spectrum Disorders (ADHD and Autism), Schizophrenia, Depression, and Anxiety. We propose that these findings could have heuristic therapeutic targeting potential and, as such, require intensive further clinical support.

## AUTHORS’ CONTRIBUTIONS

The authors confirm their contribution to the paper as follows: study conception and design: K.B and A.S., data collection: A.L., A.S., A.P., and K.M., analysis and interpretation of results: A.L., A.S. and K.B., draft manuscript: A.L., A.S. K.B. and R.B., All authors reviewed the results and approved the final version of the manuscript.

## Figures and Tables

**Fig. (1):**
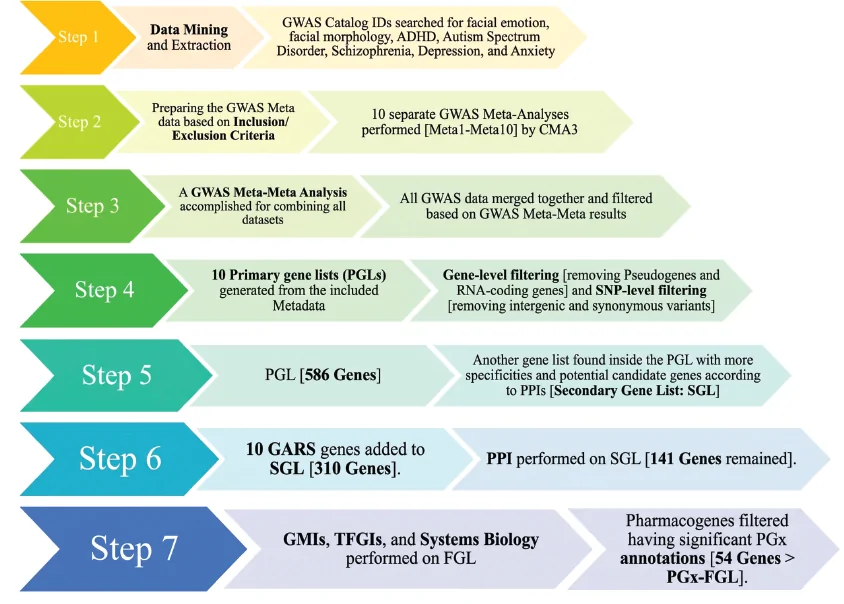
This figure presents a flowchart outlining the strategy of analysis employed in the current study. Abbreviations are as follows: GWAS: Genome-Wide Association Studies; CMA3: Comprehensive Meta Analysis 3; PGL: Primary Gene List; SGL: Secondary Gene List; FGL: Final Gene List; PPIs: Protein-Protein Interactions; GMIs: Gene-miRNA Interactions; and TFGIs: Transcription Factor-Gene Interactions.

**Fig. (2):**
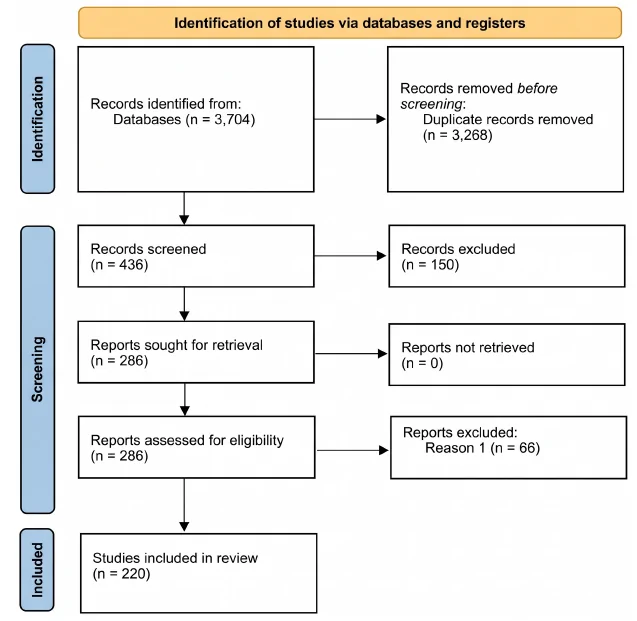
PRISMA flow diagram for systematic reviews and meta-analyses done in the current study. It should be noted that the whole data was obtained from 12 GWAS datasets. Each of which has its own GWA studies. Briefly, this diagram presents information about a large-scale metadata (called GWAS Meta-Meta). Reason 1 refers to the studies with a p-value higher than 5E-08.

**Fig. (3):**
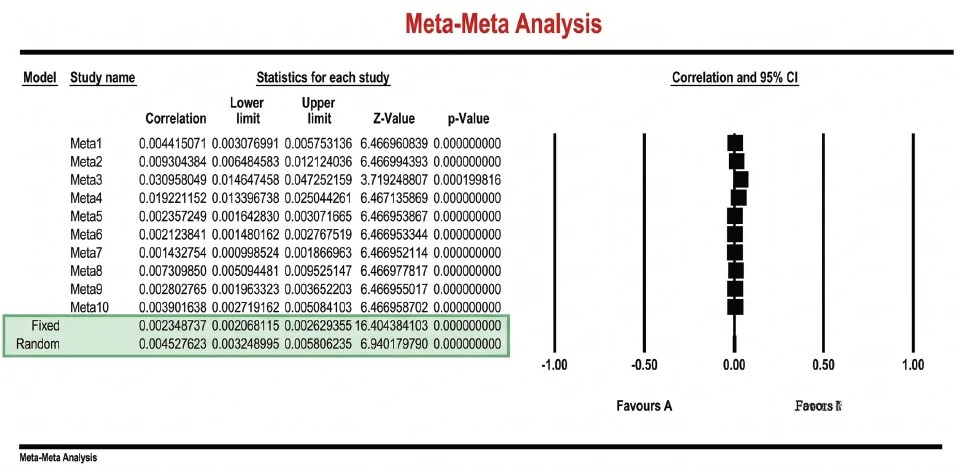
Forest Plot of GWAS Meta-Meta Analyses performed on 10 separate Meta groups, including Meta1 (Emotional Symptom Measurement), Meta2 (Facial Morphology Trait), Meta3 (Facial morphology), Meta4 (Amygdala volume), Meta5 (ADHD), Meta6 (Schizophrenia), Meta7 (Depressive Disorder), Meta8 (Anxiety), Meta9 (Anxiety Disorder), and Meta10 (Autism Spectrum Disorder). Correlation coefficient set on both Fixed and Random status with a 2-tailed and positive effect direction.

**Fig. (4):**
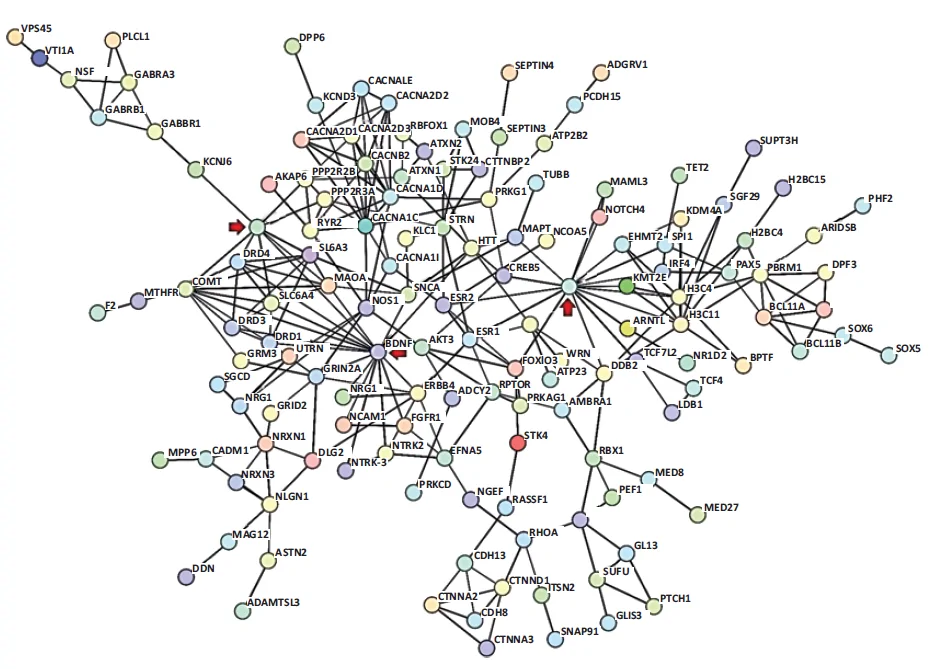
STRING-MODEL of FGL members in a completely connected PPI network. The red arrows highlight the multiple connections of *DRD2*, *BDNF, and EP300* protein-coding genes.

**Fig. (5):**
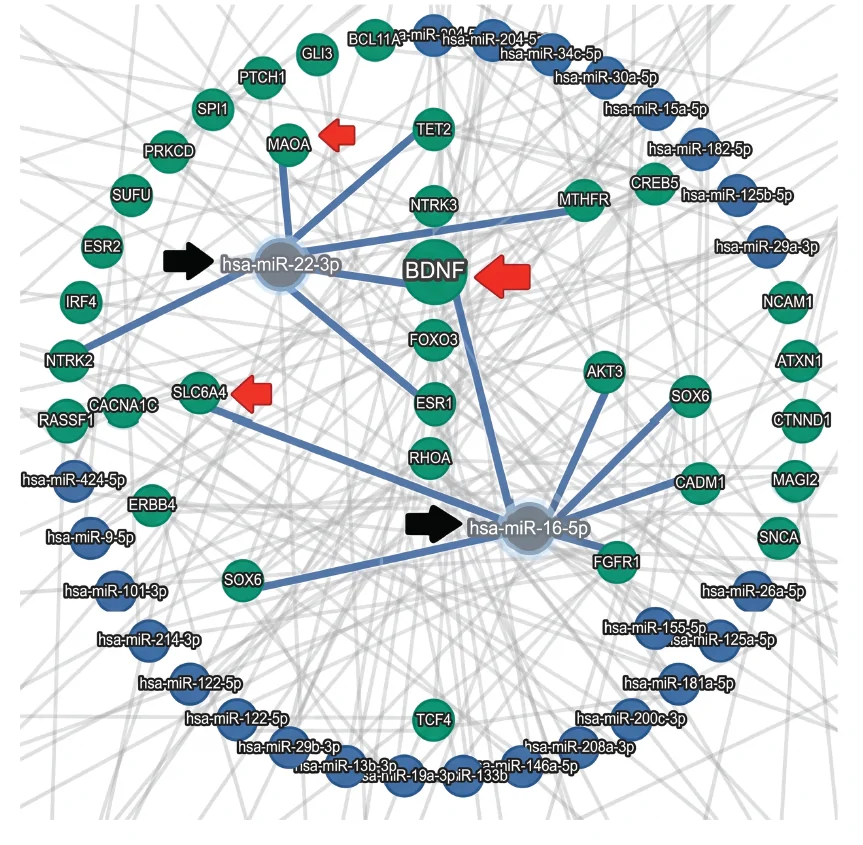
GMIs envisaged by miRTargetLink2.0 in a concentric model representing important interactions. The red arrows signify *BDNF*, *SLC6A4,* and *MAOA* genes, and the black arrows represent the key miRNAs in this network (hsa-miR16-5p and hsa-miR-22-3p). Notably, GARS genes (SLC6A4 and MAOA) can interact with *BDNF* through hsa-miR16-5p and hsa-miR-22-3p.

**Fig. (6):**
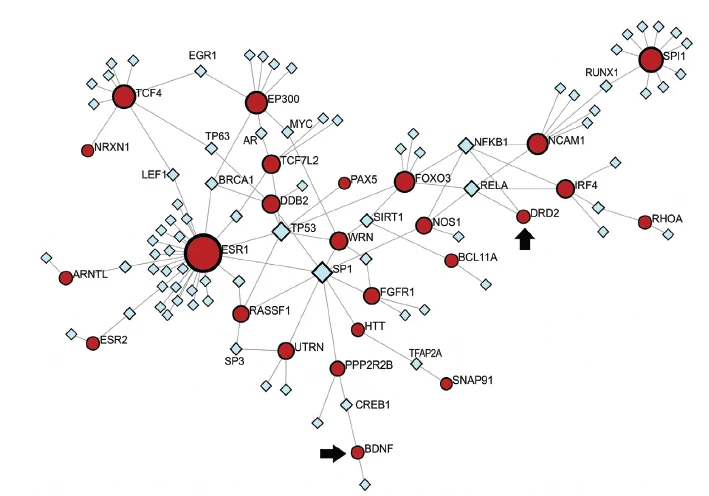
Frucherman-Reingold model of TF-Gene Interactions among the FGL members. The most interacted proteins are shown larger than others; also, blue squares refer to Transcription Factors (TFs) and black arrows highlight the *DRD2* and *BDNF* genes in this network.

**Table 1:** Tools, verifications, and references of all in-depth silico databases and systems biology methods employed in the current study.

**Level**	**Database/Tool**	**Site**	**Software (Version)**	**References**
GWAS data mining	GWAS catalog	https://www.ebi.ac.uk/gwas/home	EMBL-EBI 2024	[39]
GWAS Meta Analysis	CMA	https://meta-analysis.com/	CMA (3)	[40]
GWAS Meta-Meta Analysis	CMA	https://meta-analysis.com/	CMA (3)	[40]
PPIs	STRING-MODEL	https://string-db.org/	STRING (12.0)	[41]
GMIs	miRTarBase; miRATBase; mirDIP; miRDB	https://ccb-compute.cs.uni-saarland.de/mirtargetlink2/	miRTargetLink 2.0	[42]
GNR	TF-Gene Interactions	http://www.regnetworkweb.org/	NetworkAnalyst (3.0)	[42]
EA	Pathway Analysis	https://maayanlab.cloud/Enrichr/	Enrichr	[43]
	GO	https://maayanlab.cloud/Enrichr/	Enrichr	[43]
	DDA	https://maayanlab.cloud/Enrichr/	Enrichr	[43]
PGx	VAA	https://www.pharmgkb.org/	PharmGKB	[36, 44]

**Note:** The abbreviations are as follows: GWAS: Genome-Wide Association Study; CMA: Comprehensive Meta-Analysis; PPIs: Protein-Protein Interactions; GMIs: Gene-miRNA Interactions; GRNs: Gene Regulatory Networks; EA: Enrichment Analysis; GO: Gene Ontology; DDA: Diseases Drugs Assessment; VAA: Variant Annotation Assessment.

**Table 2:** Details of entry data (Metadata) in CMA3 from the GWAS catalog.

**Meta Group**	**Trait**	**GWAS CID**	**Unique Studies**	**Sample Size**	**Primary Unique Mapped Genes with P < E-08**
Meta1	Emotional symptom measurement	EFO_0007803	10	2,145,458	213
Meta2	Facial morphology trait	OBA_VT0003743	35	483,067	394
Meta3	Facial morphology	EFO_0004743	3	14,427	24
Meta4	Amygdala volume	OBA_2050034	3	113,180	96
Meta5	ADHD	EFO_0003888	19	7,526,398	518
Meta6	Schizophrenia	MONDO_0005090	56	9,271,590	633
Meta7	Depressive disorder	MONDO_0002050	53	20,373,022	506
Meta8	Anxiety	EFO_0005230	8	782,658	102
Meta9	Anxiety disorder	EFO_0006788	19	5,323,830	188
Meta10	ASD	EFO_0003756	14	2,747,276	303
Total	-	-	220	48,780,906	2,977

**Abbreviations:** GWAS: Genome-Wide Association Studies; CID: Catalog ID; ADHD: Attention Deficit Hyperactivity Disorder; and ASD: Autism Spectrum Disorder.

**Table 3:** Pathway analysis of FGL members according to KEGG and Reactome databases.

**Index**	**Name**	**P-value**	**q-value**	**OR**
Reactome	Neuronal system	1.15E-17	8.21E-15	11.44
Reactome	Transmission across chemical synapses	3.03E-16	1.09E-13	13.84
KEGG	Dopaminergic synapse	1.23E-15	2.41E-13	21.79
KEGG	Arrhythmogenic right ventricular cardiomyopathy	2.33E-13	2.28E-11	28.33
KEGG	cAMP signaling pathway	2.88E-12	1.88E-10	12.58
KEGG	Adrenergic signaling in cardiomyocytes	4.87E-11	2.39E-09	14.62
KEGG	MAPK signaling pathway	2.93E-10	1.15E-08	9.02
KEGG	Hypertrophic cardiomyopathy	7.78E-10	2.54E-08	18.87
KEGG	Pathways in cancer	1.04E-09	2.91E-08	6.26
KEGG	Dilated cardiomyopathy	1.48E-09	3.46E-08	17.55
KEGG	Calcium signaling pathway	1.65E-09	3.46E-08	9.58
KEGG	Cocaine addiction	1.77E-09	3.46E-08	29.07
Reactome	PI3K AKT Signaling in cancer	3.39E-10	8.10E-08	16.89
Reactome	Signal transduction	1.48E-09	2.52E-07	3.26
Reactome	Generic transcription pathway	1.76E-09	2.52E-07	4.2
Reactome	Diseases of signal transduction by Growth factor receptors and second messengers	3.10E-09	3.70E-07	6.57
Reactome	Constitutive signaling by aberrant PI3K in cancer	7.02E-09	7.20E-07	18.23
Reactome	Negative regulation of the PI3K AKT network	1.12E-08	8.85E-07	13.96
Reactome	Dopamine receptors	1.18E-08	8.85E-07	579.8
Reactome	Presynaptic depolarization and calcium channel opening	1.23E-08	8.85E-07	104.26

**Note:** Q-value and OR refer to Adjusted p-value and Odds Ratio, respectively.

**Table 4:** Gene Ontology (GO) analysis of candidate genes (FGL) in biological, cellular, and molecular processes.

**Index**	**Name**	**P-value**	**q-value**	**OR**
**GO Cellular Component**	**Calcium Channel Complex (GO:0034704)**	**2.46E-09**	**2.07E-07**	**27.72**
GO Cellular Component	Neuron Projection (GO:0043005)	2.59E-09	2.07E-07	5.91
GO Cellular Component	Voltage-Gated Calcium Channel Complex (GO:0005891)	9.49E-09	5.06E-07	32.37
GO Cellular Component	Axon (GO:0030424)	4.58E-08	1.83E-06	8.92
**GO Molecular Function**	**Voltage-Gated Calcium Channel Activity (GO:0005245)**	**7.83E-09**	**2.06E-06**	**33.41**
GO Cellular Component	Dendrite (GO:0030425)	1.00E-07	3.21E-06	7.42
**GO Biological Process**	**Dopamine Metabolic Process (GO:0042417)**	**2.78E-09**	**4.11E-06**	**67.85**
GO Biological Process	Prepulse Inhibition (GO:0060134)	1.18E-08	8.72E-06	579.8
GO Biological Process	Cell Junction Assembly (GO:0034329)	4.01E-08	1.51E-05	14.65
GO Biological Process	Negative Regulation of Transcription by RNA Polymerase II (GO:0000122)	4.56E-08	1.51E-05	4.71
GO Biological Process	Calcium Ion Transmembrane Import Into Cytosol (GO:0097553)	5.09E-08	1.51E-05	18.04
GO Biological Process	Regulation of Synaptic Transmission, Glutamatergic (GO:0051966)	7.65E-08	1.72E-05	23
GO Biological Process	Regulation of Dopamine Uptake Involved in Synaptic Transmission (GO:0051584)	8.15E-08	1.72E-05	193.25
GO Biological Process	Negative Regulation of DNA-templated Transcription (GO:0045892)	1.45E-07	2.62E-05	3.94
GO Biological Process	Positive Regulation of Intracellular Signal Transduction (GO:1902533)	1.67E-07	2.62E-05	4.73
GO Biological Process	Synapse Assembly (GO:0007416)	1.83E-07	2.62E-05	15.06
GO Cellular Component	SWI/SNF Complex (GO:0016514)	1.68E-06	4.48E-05	30.38
GO Molecular Function	Calcium Channel Activity (GO:0005262)	4.26E-07	0.000056	13.36
GO Molecular Function	Voltage-Gated Monoatomic Cation Channel Activity (GO:0022843)	9.92E-07	8.69E-05	15.2
GO Molecular Function	High Voltage-Gated Calcium Channel Activity (GO:0008331)	1.61E-06	0.000106	64.4
GO Cellular Component	Catenin Complex (GO:0016342)	4.25E-05	0.000971	24.13
GO Molecular Function	Kinase Binding (GO:0019900)	3.04E-05	0.001599	4.29
GO Cellular Component	Glutamatergic Synapse (GO:0098978)	8.53E-05	0.001705	9.15
GO Cellular Component	Postsynaptic Density (GO:0014069)	0.000165	0.002936	6.56
GO Molecular Function	Protein Kinase Binding (GO:0019901)	9.33E-05	0.004088	3.82
GO Cellular Component	Intracellular Membrane-Bounded Organelle (GO:0043231)	0.00027	0.004316	1.86
GO Molecular Function	Sequence-Specific Double-Stranded DNA Binding (GO:1990837)	0.000141	0.005304	3.3
GO Molecular Function	Transmitter-Gated Monoatomic Ion Channel Activity (GO:0022824)	0.000362	0.0119	13.15
GO Molecular Function	Transmembrane Receptor Protein Tyrosine Kinase Activity (GO:0004714)	0.000424	0.01239	12.58
GO Molecular Function	PDZ Domain Binding (GO:0030165)	0.000748	0.01781	10.71

**Note:** Q-value and OR mean adjusted p-value and Odds Ratio.

**Table 5:** Disease-Drug Assessments (DDAs) of FGL members according to DisGeNET and GeDiPNeT databases.

**Index**	**Name**	**P-value**	**q-value**	**OR**
GeDiPNet	Schizophrenia	8.51E-50	8.76E-47	15.94
GeDiPNet	Mental Depression	2.20E-40	1.13E-37	14.14
DisGeNET	Schizophrenia	4.15E-36	1.65E-32	10.15
DisGeNET	Autism Spectrum Disorders	3.50E-31	6.96E-28	15.47
GeDiPNet	Mood Disorder	6.69E-29	2.30E-26	23.23
GeDiPNet	Bipolar Disorder	1.24E-26	3.18E-24	12
DisGeNET	Autistic Disorder	5.82E-25	7.72E-22	11.52
GeDiPNet	Development Disorder	1.90E-20	3.92E-18	21.04
DisGeNET	Bipolar Disorder	1.13E-20	1.12E-17	8.8
DisGeNET	Intelligence	1.48E-19	1.17E-16	18.94
DisGeNET	Nicotine Dependence	6.62E-19	4.39E-16	27.27
GeDiPNet	Anxiety Disorder	2.78E-18	4.77E-16	12.18
DisGeNET	Mood Disorders	1.09E-18	6.20E-16	13.55
DisGeNET	Depressive disorder	1.28E-18	6.38E-16	8.61
DisGeNET	MAJOR AFFECTIVE DISORDER 4	2.02E-18	8.04E-16	41.36
DisGeNET	MAJOR AFFECTIVE DISORDER 6	2.02E-18	8.04E-16	41.36
GeDiPNet	Autism	1.07E-14	1.58E-12	7.66
GeDiPNet	Manic Disorder	8.65E-12	1.11E-09	24.63
GeDiPNet	Atrial Fibrillation	2.34E-11	2.68E-09	10.83
GeDiPNet	Breast Carcinoma	1.49E-09	1.53E-07	4.92

**Note:** Q-value stands for adjusted p-value, and OR shows Odds Ratio.

**Table 6:** Results of CpG site identification for potential CpG-SNPs related to PGx-FGL members.

**Gene**	**CpG-SNP**	**Loss/Gain**
*DRD1*	rs4532	Gain
*MTHFR*	rs1801133	Loss
*-*	rs45603435	Gain
*COMT*	rs4680	Loss
*DRD2*	rs2734837	Loss
*-*	rs1800497	Loss
*-*	rs4936272	Gain
*BDNF*	rs6265	Loss
*TCF7L2*	rs34872471	Gain
*CACNA1C*	rs758117	Loss
*-*	rs2238044	Gain
*-*	rs12823424	Gain
*-*	rs10774037	Loss
*-*	rs2007044	Gain
*-*	rs2159100	Loss
*-*	rs11062161	Gain
*-*	rs4298967	Gain
*-*	rs1024582	Gain
*-*	rs4765914	Gain
*-*	rs1205787230	Gain
*GRM3*	rs35274762	Gain
*-*	rs6943762	Gain
*NRXN1*	rs13031157	Gain
*CACNB2*	rs7893279	Gain
*-*	rs6482423	Gain
*-*	rs17691888	Loss
*KCNJ6*	rs2154556	Gain
*-*	rs1787402	Loss
*F2*	rs61884307	Gain
*ARID5B*	rs713240	Loss
*-*	rs72833340	Gain
*BCL11A*	rs7599488	Loss
*CDH13*	rs62035176	Loss
*-*	rs78600318	Gain
*ASTN2*	rs4629949	Loss
*-*	rs151323364	Loss
*-*	rs35623509	Gain
*CACNA2D3*	rs17054293	Gain
*ESR2*	rs915057	Gain
*-*	rs7146434	Gain
*-*	rs1152578	Gain
*CACNA1E*	rs4652676	Loss
*FOXO3*	rs9398171	Gain
*-*	rs4945816	Loss
*-*	rs2153960	Loss
*GRID2*	rs1972860	Loss
*NRG1*	rs2347498	Loss
*RPTOR*	rs11651818	Gain
*-*	rs8066384	Gain
*ADGRV1*	rs11959408	Loss
*CACNA1D*	rs13080668	Gain
*-*	rs7649275	Gain
*CACNA1I*	rs5750853	Loss
*-*	rs5995756	Gain
*-*	rs8139773	Gain
*-*	rs926231	Loss
*CTNNA2*	rs72927416	Gain
*CTNNA3*	rs1993849	Loss
*DLG2*	rs10898240	Loss
*-*	rs1864774	Loss
*KLC1*	rs35229468	Loss
*-*	rs4906338	Gain
*-*	rs4906349	Gain
*MAML3*	rs11100449	Loss
*-*	rs1455480	Loss
*NCAM1*	rs12224032	Loss
*-*	rs12574893	Loss
*-*	rs3802850	Gain
*-*	rs7110863	Gain
*PRKG1*	rs61847303	Loss
*RBFOX1*	rs12923795	Gain
*-*	rs13332522	Loss
*-*	rs2191130	Gain
*-*	rs4494563	Loss
*-*	rs4786809	Loss
*-*	rs7198928	Gain
*-*	rs8063603	Loss
*RHOA*	rs7623659	Loss
*VTI1A*	rs12245585	Loss
*-*	rs1961513	Gain

## Data Availability

All data generated or analyzed during this study are included in this published article.

## LIST OF ABBREVIATIONS

PGx: Pharmacogenomics
GWAS: Genome-Wide Association Studies
SGL: Secondary Gene List
PPIs: Protein-Protein Interactions
FGL: Final Gene List
ADHD: Attention Deficit Hyperactivity Disorder
IBM: International Business Machines
FER: Facial Emotion Recognition
NIBS: Non-Invasive Brain Stimulation
dlPFC: dorsolateral Prefrontal Cortex
vmPFC: ventromedial Prefrontal Cortex
rTPJ: right Temporoparietal Junction
pSTS: posterior Superior Temporal Sulcus
pre-SMA: pre-Supplementary Motor Area
ASD: Autism Spectrum Disorder
FAR: Facial Affect Recognition
MFERT: Morphed Facial Emotion Recognition Task
RMET: Reading the Mind in the Eyes Task
BFRT: Benton Face Recognition Test
ERPs: Event-Related Potentials
ER: Emotion Recognition
STS: Superior Temporal Sulcus
PFC: Prefrontal Cortex
ASC: Autism Spectrum Condition
HL: High Likelihood
SRS: Social Responsiveness Scale
PGL: Primary Gene List
SGL: Secondary Gene List
FGL: Final Gene List
ES: Effect Size
OR: Odds Ratio
CI: Confidence Interval
SNP: Single Nucleotide Polymorphism
CMA: Comprehensive Meta-Analysis
PreSGL: Pre-Secondary Gene List
TFGIs: TF-Gene Interactions
PA: Pathway Analysis
KEGG: Kyoto Encyclopedia of Genes and Genomes
GO: Gene Ontology
BDNF: Brain-Derived Neurotrophic Factor
TrkB: Tyrosine kinase receptor B
PTSD: Post-Traumatic Stress Disorder
CSF: Cerebrospinal fluid

## STANDARDS OF REPORTING

PRISMA guidelines were followed.

## SUPPLEMENTARY MATERIAL

PRISMA Checklist is available on the publisher's website along with the published article.
