# Supplementary material for: Pharmacogenomic-based GWAS Meta-Analyses Coupled with Genetic and Epigenetic Liability Testing Connects Facial and Emotional Recognition Systems to Spectrum Disorders, Schizophrenia, Depression, and Anxiety Disorders
Source: Curr Pharm Biotechnol. 2026 Mar 9;27(10):1243–62. doi: 10.2174/0113892010431102260107110422 (PMC13698029; doi:10.2174/0113892010431102260107110422)
Supplement: Supplementary file 1 [file CPB-27-10-1243_SD1.pdf]

Supplementary Material

Pharmacogenomic-Based GWAS Meta-Analyses Coupled with Genetic and Epigenetic Liability Testing Connects Facial and Emotional Recognition Systems to Spectrum Disorders, Schizophrenia, Depression, and Anxiety

Kenneth Blum[1-4,**], Alexander P.L. Lewandrowski[3,5], Alireza Sharafshah[6], Albert Pinhasov[1], Kavya Mohankumar[3], Mark S. Gold[7], Brian Fuehrlein[8], Igor Elman[9], Catherine Dennen[10], Panayotis K. Thanos[11], Abdalla Bowirrat[1], David Baron[2,12], Edward J. Modestino[13], Nicole Jafari[14], Foojan Zeine[15], Keerthy Sunder[2,16], Milan T. Makale[17], Morgan P. Lorio[18], Jag Khalsa[19], Sergio Luis Schmidt[20], Rossano Kepler Alvim Fiorelli[20], Aviv Weinstein[21], Marco Lindenau[3], Kai-Uwe Lewandrowski[20,22-24], Óscar L. Alves[25], Shaurya Mahajan[3], Yatharth Mahajan[3] and Rajendra D. Badgaiyan[26,**]

[1]Department of Molecular Biology, Adelson School of Medicine, Ariel University, Ariel 40700, Israel; [2]Division of Addiction Research & Education, Center for Sports, Exercise, Psychiatry, Western University Health Sciences, Pomona, CA 91766-1854, USA; [3]The Kenneth Blum Behavioral & Neurogenetic Institute, Austin, TX 78701, USA; [4]Institute of Psychology, ELTE Eötvös Loránd University, H-1117 Budapest, Hungary; [5]Department of Biological Sciences, Dornsife College of Letters, Arts and Sciences, 3616 Trousdale Pkwy, Los Angeles, CA 90089, USA; [6]Cellular and Molecular Research Center, School of Medicine, Guilan University of Medical Sciences, Rasht 4144666949, Iran; [7]Department of Psychiatry, Washington University School of Medicine, St. Louis, MO 63110, USA; [8]Department of Psychiatry, Yale University, New Haven, CT, 06520, USA; [9]Cambridge Health Alliance, Harvard Medical School, Cambridge, MA, 02139, USA; [10]Department of Family Medicine, Jefferson Health Northeast, Philadelphia, PA, 19145, USA; [11]Department of Pharmacology and Toxicology, State University of New York, SUNY, Buffalo, NY 14215, USA; [12]Department of Psychiatry, Stanford University School of Medicine, Palo Alto, CA 94305, USA; [13]Brain & Behavior Laboratory, Department of Psychology, Curry College, Milton, MA 02186, USA; [14]Department of Applied Clinical Psychology, The Chicago School of Professional Psychology, Los Angeles, CA 60601, USA; [15]Department of Health Science, California State University at Long Beach, Long Beach, CA 90815, USA; [16]Department of Psychiatry, University of California, UC Riverside School of Medicine, Riverside, CA 92521, USA; [17]Department of Radiation Medicine and Applied Sciences, University of California San Diego, La Jolla, CA 92093, USA; [18]Advanced Orthopedics, Altamonte Springs, FL 32701, USA; [19]Medical Consequences of Drug Abuse and Infections Branch, National Institute on Drug Abuse, National Institutes of Health, Bethesda, Maryland, MD 20852, USA; [20]Programa de Pós-Graduação em Neurologia, Universidade Federal do Estado do Rio de Janeiro, Rio de Janeiro 20270-004, Brazil; [21]Department of Behavioral Sciences, Ariel University, Ariel, 40700, Israel; [22]Division of Personalized Medicine, Center for Advanced Spine Care of Southern Arizona, Tucson, AZ 85712, USA; [23]Department of Orthopaedics, Fundación Universitaria Sanitas, Bogotá 110131, Colombia; [24]Department of Orthopaedic Surgery, University of Arizona, School of Medicine, Tucson, AZ 85724, USA; [25]Department of Neurosurgery, Hospital Lusíadas, Porto, Portugal; [26]Department of Psychiatry, Texas Tech University Health Sciences, School of Medicine, Midland, TX 79430, USA

Section and Topic	Item #	Checklist item	Location where item is reported
**TITLE**			
Title	1	Identify the report as a systematic review.	Page 1
**ABSTRACT**			
Abstract	2	See the PRISMA 2020 for Abstracts checklist.	Page 3
**INTRODUCTION**			
Rationale	3	Describe the rationale for the review in the context of existing knowledge.	Page 3
Objectives	4	Provide an explicit statement of the objective(s) or question(s) the review addresses.	Page 3-4
**METHODS**			
Eligibility criteria	5	Specify the inclusion and exclusion criteria for the review and how studies were grouped for the syntheses.	Page 7
Information sources	6	Specify all databases, registers, websites, organisations, reference lists and other sources searched or consulted to identify studies. Specify the date when each source was last searched or consulted.	Page 7-8
Search strategy	7	Present the full search strategies for all databases, registers and websites, including any filters and limits used.	Page 7
Selection process	8	Specify the methods used to decide whether a study met the inclusion criteria of the review, including how many reviewers screened each record and each report retrieved, whether they worked independently, and if applicable, details of automation tools used in the process.	Page 7-8

Section and Topic	Item #	Checklist item	Location where item is reported
Data collection process	9	Specify the methods used to collect data from reports, including how many reviewers collected data from each report, whether they worked independently, any processes for obtaining or confirming data from study investigators, and if applicable, details of automation tools used in the process.	Page 8-9
Data items	10a	List and define all outcomes for which data were sought. Specify whether all results that were compatible with each outcome domain in each study were sought (e.g. for all measures, time points, analyses), and if not, the methods used to decide which results to collect.	Page 10-11
	10b	List and define all other variables for which data were sought (e.g. participant and intervention characteristics, funding sources). Describe any assumptions made about any missing or unclear information.	Page 10
Study risk of bias assessment	11	Specify the methods used to assess risk of bias in the included studies, including details of the tool(s) used, how many reviewers assessed each study and whether they worked independently, and if applicable, details of automation tools used in the process.	Page 12
Effect measures	12	Specify for each outcome the effect measure(s) (e.g. risk ratio, mean difference) used in the synthesis or presentation of results.	Page 14
Synthesis methods	13a	Describe the processes used to decide which studies were eligible for each synthesis (e.g. tabulating the study intervention characteristics and comparing against the planned groups for each synthesis (item #5)).	Page 13
	13b	Describe any methods required to prepare the data for presentation or synthesis, such as handling of missing summary statistics, or data conversions.	Page 13
	13c	Describe any methods used to tabulate or visually display results of individual studies and syntheses.	Page 14
	13d	Describe any methods used to synthesize results and provide a rationale for the choice(s). If meta-analysis was performed, describe the model(s), method(s) to identify the presence and extent of statistical heterogeneity, and software package(s) used.	Page 15
	13e	Describe any methods used to explore possible causes of heterogeneity among study results (e.g. subgroup analysis, meta-regression).	Page 15
	13f	Describe any sensitivity analyses conducted to assess robustness of the synthesized results.	Page 15
Reporting bias assessment	14	Describe any methods used to assess risk of bias due to missing results in a synthesis (arising from reporting biases).	Page 15
Certainty assessment	15	Describe any methods used to assess certainty (or confidence) in the body of evidence for an outcome.	Page 15
**RESULTS**			
Study selection	16a	Describe the results of the search and selection process, from the number of records identified in the search to the number of studies included in the review, ideally using a flow diagram.	Page 14
	16b	Cite studies that might appear to meet the inclusion criteria, but which were excluded, and explain why they were excluded.	Page 14
Study characteristics	17	Cite each included study and present its characteristics.	Page 16-20
Risk of bias in studies	18	Present assessments of risk of bias for each included study.	Page 21
Results of individual studies	19	For all outcomes, present, for each study: (a) summary statistics for each group (where appropriate) and (b) an effect estimate and its precision (e.g. confidence/credible interval), ideally using structured tables or plots.	Page 22-23
Results of syntheses	20a	For each synthesis, briefly summarise the characteristics and risk of bias among contributing studies.	Page 24
	20b	Present results of all statistical syntheses conducted. If meta-analysis was done, present for each the summary estimate and its precision (e.g. confidence/credible interval) and measures of statistical heterogeneity. If comparing groups, describe the direction of the effect.	Page 25-27
	20c	Present results of all investigations of possible causes of heterogeneity among study results.	Page 27
	20d	Present results of all sensitivity analyses conducted to assess the robustness of the synthesized results.	Page 28
Reporting biases	21	Present assessments of risk of bias due to missing results (arising from reporting biases) for each synthesis assessed.	Page 29
Certainty of evidence	22	Present assessments of certainty (or confidence) in the body of evidence for each outcome assessed.	Page 30
**DISCUSSION**			
Discussion	23a	Provide a general interpretation of the results in the context of other evidence.	Page 30
	23b	Discuss any limitations of the evidence included in the review.	Page 30
	23c	Discuss any limitations of the review processes used.	Page 30

Section and Topic	Item #	Checklist item	Location where item is reported
	23d	Discuss implications of the results for practice, policy, and future research.	Page 30-32
**OTHER INFORMATION**			
Registration and protocol	24a	Provide registration information for the review, including register name and registration number, or state that the review was not registered.	-
	24b	Indicate where the review protocol can be accessed, or state that a protocol was not prepared.	-
	24c	Describe and explain any amendments to information provided at registration or in the protocol.	-
Support	25	Describe sources of financial or non-financial support for the review, and the role of the funders or sponsors in the review.	Page 32
Competing interests	26	Declare any competing interests of review authors.	Page 32
Availability of data, code and other materials	27	Report which of the following are publicly available and where they can be found: template data collection forms; data extracted from included studies; data used for all analyses; analytic code; any other materials used in the review.	N/A

From: Page MJ, McKenzie JE, Bossuyt PM, Boutron I, Hoffmann TC, Mulrow CD, et al. The PRISMA 2020 statement: an updated guideline for reporting systematic reviews. BMJ 2021;372:n71. doi: 10.1136/bmj.n71

For more information, visit: http://www.prisma-statement.org/
